# Imaging CRISPR-edited CAR-T cell therapies with optical and positron emission tomography reporters

**DOI:** 10.7150/thno.119013

**Published:** 2026-01-01

**Authors:** Rafael Enrique Sanchez-Pupo, John Joseph Kelly, Nourhan Shalaby, Ying Xia, Francisco Manuel Martinez-Santiesteban, Jasmine Lau, Ivy Elizabeth Verriet, Matthew Stefan Fox, Justin Wesley Hicks, Jonathan Dale Thiessen, John Andrew Ronald

**Affiliations:** 1 Robarts Research Institute, University of Western Ontario, London, ON, Canada.; 2 Dept. of Medical Biophysics, University of Western Ontario, London, ON, Canada.; 3 Lawson Health Research Institute, London, ON, Canada.; 4 Dept. of Medical Physics, CancerCare Manitoba, Winnipeg, MB, Canada.; 5 Dept. of Radiology, University of Manitoba, Winnipeg, MB, Canada.; 6 Dept. of Physics and Astronomy, University of Western Ontario, London, ON, Canada.

**Keywords:** CAR-T, cell tracking, PET, CRISPR-based genome editing, immunotherapy

## Abstract

**Rationale:** Chimeric antigen receptor (CAR) T cell therapies have shown remarkable success in treating hematological cancers and are increasingly demonstrating potential for solid tumors. CRISPR-based genome editing offers a promising approach to enhance CAR-T cell potency and safety, yet challenges such as inefficient tumor homing and toxicities in normal tissues, limit broader adoption. Advanced imaging technologies, including bioluminescence imaging (BLI) and positron emission tomography (PET), provide real-time visualization of CAR-T cell behavior *in vivo*. Here, we developed *Trackable Reporter Adaptable CRISPR-Edited CAR* (tRACE-CAR) T cells, a modular system for site-specific integration of CARs and imaging reporters.

**Methods:** The luciferase reporter AkaLuciferase (AkaLuc) or the human sodium iodide symporter (NIS) was cloned downstream of the CAR in adeno-associated virus (AAV) donors for imaging. CAR-reporter cassettes were inserted into the T-cell receptor α constant locus of primary human T cells via CRISPR editing and AAV transduction. Editing efficiency was assessed by flow cytometry. *In vitro* cytotoxicity was evaluated across multiple effector-to-target ratios. *In vivo*, BLI and PET imaging were used for tracking CAR-T cells in tumor-bearing immunodeficient mice.

**Results:** T cell receptor (TCR) knockout efficiency exceeded 85% and CAR expression reached 70-80%. Reporter-engineered CAR-T cells exhibited significant cytotoxicity and outperformed naïve T cells. *In vivo*, AkaLuc BLI and ^18^F-tetrafluoroborate PET enabled non-invasive tracking of viable CAR-T cells. Administration route (intravenous, peritumoral, or intraperitoneal) significantly influenced CAR-T cell distribution and therapeutic effectiveness.

**Conclusion:** tRACE-CAR enables precise optical and PET tracking of CRISPR-edited CAR-T cells in models of leukemia and ovarian cancer, allowing dynamic, non-invasive monitoring of cell distribution in both tumors and off-target tissues. This imaging-enabled platform could lead to more personalized, effective CRISPR-edited CAR cell therapies.

## Introduction

Chimeric antigen receptors (CARs) are synthetic proteins designed to enhance the antigen-specific recognition and attack of cancer cells by immune cells such as T lymphocytes 1,2. CAR-T cell therapy is now considered a standard treatment for patients with relapsed or refractory B-cell lymphomas, B-cell acute lymphoblastic leukemia, and multiple myeloma 3,4. Recent successes in patients with brain cancer (e.g., neuroblastoma) have renewed excitement about the applicability of CAR-T cells for solid tumors 5. However, significant challenges remain, like patient non-response/relapse and adverse effects such as cytokine release syndrome (CRS), immune effector cell-associated neurotoxicity syndrome (ICANS), and on-target/off-tumor harm of healthy cells 4,6-9. In solid tumors, poor CAR cell trafficking, antigen heterogeneity, and immunosuppressive microenvironments can also reduce therapy effectiveness 10.

To date, clinically approved CAR-T products utilize viral transduction to integrate transgenes into the T cell genome 11. Lentiviral vectors tend to integrate into transcriptionally active regions of the genome, which has raised concerns about insertional oncogenesis 12,13 and a potential association with T-cell malignancies 14,15, prompting an FDA boxed warning in 2023 16. While causality is unclear 17-19, the risk of insertional mutagenesis with viral vectors highlights the need for improved strategies to enhance CAR-T cell efficacy and safety, as well as robust technologies to monitor CAR-T cell dynamics *in vivo* and better understand treatment outcomes and adverse effects.

To improve the therapeutic index of CAR-T cells, many groups have used clustered regularly interspaced short palindromic repeats (CRISPR)-based genome editing to precisely knock-out certain endogenous genes, knock-in transgenes of interest at specific loci, or both 20. In 2017, a landmark study used CRISPR to knock-in an anti-CD19 CAR transgene into the T-cell receptor α constant (*TRAC*) locus of human T cells, disrupting T cell receptor (TCR) assembly and enhancing therapeutic efficacy against B-cell leukemia in mice, compared to lentiviral-engineered cells 21. More recent studies have refined this approach, achieving higher HDR-mediated integration efficiencies at the *TRAC* locus using optimized CRISPR/Cas systems and donor designs 22. Clinical trials using CRISPR-based targeting at the *TRAC* loci and/or other loci aimed at making more efficient, safer, and cost-effective CAR cell products are emerging 23-26.

As novel CAR cells are developed and translated, complementary technologies are essential for improved post-infusion monitoring. Current methods assessing circulating CAR-T cells or their products fail to fully capture their activity within tumors or other tissues 27. Preclinical and clinical reporter gene imaging technologies can offer non-invasive, spatiotemporal insights into CAR cell migration, proliferation, and persistence after infusion. For instance, orthogonal reporters for bioluminescence imaging (BLI) have allowed for highly sensitive tracking of both CAR cells and cancer cells in individual animals 28,29. Clinically relevant reporters for positron emission tomography (PET) derived from viruses 30,31, bacteria 32, humans 27,29,33-38 or artificially engineered surface receptors 39 have demonstrated the ability to visualize lentiviral-engineered CAR-T and CAR-Natural Killer (CAR-NK) cells in various preclinical cancer models, and pioneering clinical studies using a virally-derived PET reporter firmly established the ability to track CAR-T cells in high-grade glioma patients 40,41. Comprehensive reviews have underscored the growing integration of multimodal molecular imaging technologies into CAR-T development and evaluation pipelines 42,43.

To leverage upon CRISPR-edited CAR cells and BLI/PET reporter-based cell tracking, this research focused on developing an efficient, site-directed, modular system to produce **T**rackable **R**eporter **A**daptable **C**RISPR-**E**dited **CAR** (tRACE-CAR) T cells. We validated this technology by editing T cells at the *TRAC* locus with different CARs and imaging reporter genes, allowing us to visualize CAR-T cell accumulation at both on- and off-tumor sites in models of B cell leukemia and ovarian cancer. The tRACE-CAR system is an affordable solution that can be adapted to any genomic locus, offering relatively high editing efficiency and the ability to track the edited cells in preclinical models (BLI/PET) and patients (PET). Our system has the potential to improve the safety and effectiveness of CAR-T therapies and provide real-time *in vivo* information on CAR-T fate. These insights could lead to improved understanding of tumor response and off-target effects, supporting next-generation CAR development and improved patient management and therapeutic outcomes.

## Material and Methods

### Cell lines and culture

NALM6 cells (ATCC: clone G5, #CRL-3273) were grown in RPMI-1640 Medium supplemented with 10% fetal bovine serum (FBS) and 1% antibiotic-antimycotic (A/A) (Thermo Fisher Scientific). HER2-expressing SKOV3.ip1 (RRID:CVCL_0C84) ovarian cancer cells (kindly provided by Dr. Trevor Shepherd, London Regional Cancer Program, University of Western Ontario, ON) were cultured in McCoy's 5A medium (Thermo Fisher Scientific) containing 10% FBS and 1% A/A. Cells were transduced as previously reported 44 with home-made lentiviral vectors to express dTomato (dT) and Firefly luciferase (NALM6-dt-Luc2 and SKOV3-dT-Luc2) or zsGreen and Antares (SKOV3-zsG-Antares). Upon transduction, positive cell populations were sorted based on the fluorescence of dT, zsG, or Antares with a BD FACSAria III cell sorter (BD Biosciences) in the London Regional Flow Cytometry Facility at Robarts Research Institute, University of Western Ontario. All cells were grown in a humidified incubator at 37 °C and 5% CO_2_ and were routinely tested for mycoplasma contamination with the MycoAlert PLUS Mycoplasma Detection Kit (Lonza).

### T cell activation and culture

Human peripheral blood mononuclear cells (PBMCs) (STEMCELL Technologies, Cat# 70025.1) were stimulated with Dynabeads Human T-Activator CD3/CD28 (Thermo Fisher Scientific) at a bead-to-cell ratio of 1:1. After 3 days, Dynabeads were removed using an EasySep magnet (STEMCELL Technologies). Activated T cells were cultured in ImmunoCult-XF T cell expansion medium (STEMCELL Technologies) containing 1% A/A and 200 U/mL rhIL-2 (kindly provided by the BRB Preclinical Biologics Repository, Frederick National Laboratory for Cancer Research, USA). The cell culture medium was changed every 2-3 days and cells were maintained at a density of 1 x 10^6^ cells/mL in a humidified incubator at 37 °C, 5% CO_2_.

### Adeno-associated virus vector design and production

Adeno-associated virus donor vectors (pAAV) containing AAV serotype 2 inverted terminal repeats were designed so that transgene expression was driven off the endogenous *TRAC* promoter by adding a splice acceptor site (SA) and a self-cleaving T2A peptide in frame with the *TRAC* locus, as described by 21. A CAR-Imaging reporter gene cassette was cloned immediately after the T2A cleavage sequence, flanked by 200 bp or 600 bp homologous arms (HA sequence) specific to the first exon of the *TRAC* gene. The donor cassette comprised a cDNA encoding a CD19-specific CAR (herein referred to as CD19CAR, kindly provided by Dr's Holt and Nelson, University of Victoria, Victoria, BC, Canada 45) or a HER2-targeting designed ankyrin repeat protein (DARPin)-based CAR (referred to as HER2CAR, kindly provided by Dr. Bramson, McMaster University, Hamilton, ON, Canada^46^) followed by a self-cleaving P2A peptide in frame with an imaging reporter gene and the bovine growth hormone (bGH) polyadenylation signal. Specifically, the imaging reporter genes cloned herein were: eGFP, for fluorescence, AkaLuc 47 for bioluminescence imaging (BLI), or the human sodium iodide symporter (NIS) 36 for PET. AAV serotype 6 (AAV6) particles were produced and purified by SignaGen Laboratories (Frederick, MD, USA) and VectorBuilder (Chicago, IL, USA).

### CRISPR/Cas9 and AAV transduction

Activated primary human T cells were nucleofected with ribonucleoprotein (RNP) complexes consisting of Alt-R® S.p. HiFi Cas9 Nuclease V3 (IDT, Cat#1081061) and synthetic *TRAC* sgRNA (5′-CAGGGUUCUGGAUAUCUGU-3′) (Synthego, USA) as per 21 using the P3 Primary Cell 4D-Nucleofector X Kit S (Lonza). To make the RNP complexes, 1 μL of sgRNA (60 μM) and 1 μL of HiFi Cas9 Nuclease (20 μM) were mixed in a 0.2 mL PCR tube (3:1 gRNA:Cas9 molar ratio) and incubated for 10 min at room temperature. 1 x 10^6^ activated T cells were pelleted and resuspended in 20 μL of P3 Primary Cell solution + Supplement 1 (Lonza) and mixed with the RNP complexes. Cells and RNPs were transferred to a 16-well Nucleocuvette Strip and nucleofected in a 4D-Nucleofector™ X Unit (Lonza) using the pre-set program E0-115 for stimulated human T cells. Following nucleofection, the cells were left to incubate at room temperature for 5 min before adding 80 μL of warm T cell culture medium and incubating for 10 min at 37 °C, 5% CO_2_. Finally, the cells were gently resuspended and transferred to a 48-well culture plate with 400 μL of the same culture media. Thirty minutes after nucleofection, rAAV6s were added to the cell culture at a multiplicity of infection of 1 × 10^5^ virions per cell (unless described otherwise). Engineered CAR-T cells were used without further purification to preserve cell viability and maintain a representative mixture of effector and memory T cell subsets which was considered appropriate for early-stage feasibility and imaging studies.

### PCR integration assays

DNeasy Blood and Tissue Kit (QIAGEN) was used to extract genomic DNA from edited T cells following the manufacturer's instructions. 150 ng of genomic DNA isolated from engineered T cells was used as the template for PCR. A reverse primer was designed to target intron 1 in the *TRAC* locus (5'-TGACTGCGTGAGACTGACTT-3'), and a forward primer in the bovine growth hormone (bGH) polyadenylation signal of the AAV donor cassette (5'-TGGGAAGACAATAGCAGGCA-3').

### Flow cytometry

Genome-editing efficiency was assessed by flow cytometry using a FACSCanto™ cytometer (BD Biosciences) 7-9 days after nucleofection and rAAV6 transduction. Immunophenotyping by flow cytometry was performed using a FACSymphony™ A1 cytometer and analyzed with FlowJo v10.10.0 for macOS. Where applicable, rhCD19-Fc-Chimera-Alexa Fluor® 647 (Bio-Techne, Cat# AFR9269) or rhErbB2/Her2-Fc-His-Alexa Fluor® 647 (Bio-Techne, Cat# AFR1129) fluorokines were used to measure surface expression of CD19CAR or HER2CAR, respectively. TCR expression was assessed using an anti-human TCRα/β PE-Cyanine7-conjugated antibody (BioLegend, Cat# 306719). CD4 and CD8 expression were evaluated using anti-human CD4 PE/Cyanine7 (BioLegend, Cat# 357409) and anti-human CD8a APC (BioLegend, Cat# 300911). SYTOX™ Blue (Thermo Fisher Scientific) was used as a viability dye to exclude dead cells. Immunophenotyping for T cell memory subsets was performed using fluorochrome-conjugated antibodies against CD3, CD4, CD8, CD45RA, and CD197 (CCR7) from the BD Horizon™ Human T Cell Backbone Panel Kit (BD Biosciences, Cat# 568263), following the manufacturer's instructions. PD-1 and CD56 expression were assessed using anti-human PD-1 (CD279)-PE (BD Biosciences, Cat# 567617) and anti-human CD56-PE (BioLegend, Cat# 318305), respectively.

### *In vitro* BLI and cytotoxicity assays

Seven days after nucleofection, CAR-T cell cytotoxicity was assessed by co-incubating CAR-T cells with firefly luciferase (Fluc)-expressing target cancer cells and measuring BLI over time. Briefly, 1.25 x 10^4^ target cells/well were plated in 96-well plates in 100 μL of serum-supplemented culture media. T cells were then added at various effector-to-target ratios (0.5:1, 1:1, 2:1, or 4:1). The plate was incubated for 18-24 h at 37 °C, 5% CO_2_. BLI was acquired with an IVIS Lumina XRMS *In Vivo* Imaging System (PerkinElmer) after adding D-luciferin (150 μg/mL, Syd Labs) to each well. Regions of interest (ROI) were drawn over each well to quantify the peak average radiance per well (photons(p)/second(s)/cm^2^/steradian(sr)). BLI data analysis was conducted using the Living Image Software (PerkinElmer). Percent target cell lysis was calculated using Equation 1:


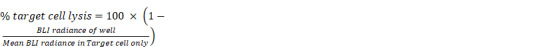

(Eq. 1)

To assess for AkaLuc reporter gene functionality, naïve or AAV-only control (AAV6-CAR-AkaLuc transduction but no CRISPR/Cas9-editing) and CAR-AkaLuc (TRAC-edited + AAV6-CAR-AkaLuc) T cells were seeded in triplicate in 96-well culture black plates and serially diluted from 2, 1, 0.5, 0.25, 0.1, 0.05 (× 10^6^) cells/well. Fifteen minutes of BLI acquisition at 37 °C was conducted after adding AkaLumine-HCl (TokeOni; Sigma-Aldrich) substrate (0.125 mmol/L, final concentration) to the T cell culture. Peak average radiance was determined from ROIs of each well and quantified as above.

### *In vitro* [^18^F]-TFB uptake

[^18^F]-TFB was synthesized as described previously 48. Naïve or CAR-NIS-expressing T cells were seeded in a 24-well dish (1 × 10^6^ cells/well), and 0.1 MBq of [^18^F]-TFB was added to each well. Cells were incubated for 45 min before centrifuging and washing the cell pellets with ice-cold PBS twice. The activity of harvested cells was measured using a gamma counter, and data was presented as net counts.

### Animal experiments and ethical approval

All animal procedures were approved by the Animal Care Committee at the University of Western Ontario (AUP protocol 2018-150) and conducted in accordance with the ARRIVE guidelines 2.0 (https://arriveguidelines.org/). Male and female NOD-scid IL2Rgamma-null (NSG) mice aged 8-13 weeks were used, sourced from an in-house colony (Dr. David Hess, Western University). Female mice were exclusively used for experiments involving SKOV3-derived ovarian cancer models due to tumor biology and anatomical relevance. Animals were housed under standardized conditions with a 12 h light/dark cycle, ambient temperature maintained between 22-24 °C, and relative humidity around 50%. Food and water were provided *ad libitum*. Mice were monitored daily for signs of distress or illness, and humane endpoints were predefined based on tumor burden, body weight loss (>20%), ulceration, or clinical signs of poor health (e.g., labored breathing, ascites, hemorrhage). Randomization was performed prior to treatment allocation, and investigators were blinded during imaging analysis and endpoint assessments. Sample sizes were determined based on prior experience and pilot studies to ensure sufficient statistical power while minimizing animal use.

### Tumor implantation and treatment models

*Leukemia Model (NALM6):* NSG mice (6-8 weeks old) received 0.5 × 10⁶ NALM6-Antares cells via tail vein injection. Tumor engraftment was confirmed by bioluminescence imaging (BLI) the following day. On Day 3 or 4, mice were injected intravenously with 1 × 10⁷ naïve or CD19CAR-AkaLuc T cells. BLI was performed at multiple time points to monitor tumor progression and CAR-T cell localization.

*Subcutaneous Ovarian Cancer Model:* SKOV3-ip1 or SKOV3-dT-Fluc cells (1 × 10⁶) were mixed 1:1 with Matrigel and injected subcutaneously into the right hind flank. Tumor growth was monitored via caliper measurements or bioluminescence, and volume was calculated using Equation 2:


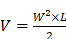

(Eq. 2)

where *W* is width and *L* is length. When tumors reached ~100 mm³ (7-14 days after implantation), mice were randomized into treatment groups. Each group received one of the following: saline, a single dose of 5 × 10⁶ naïve T cells, a single dose of 5 × 10⁶ HER2CAR-AkaLuc T cells, or three doses (each 5 × 10⁶ cells) of HER2CAR-NIS T cells administered intravenously or peritumorally. Endpoint criteria included tumor volume ≥2.0 cm³, tumor radiance ≥8 × 10⁸ p/s/cm²/sr, ulceration, or health deterioration.

*Intraperitoneal Ovarian Cancer Model:* On Day 0, 1 × 10⁵ SKOV3-dT-Fluc cells or 1 x 10^6^ SKOV3-zsG-Antares were injected intraperitoneally. Mice received one or three IP doses of saline, naïve, HER2CAR-Akaluc, HER2CAR-GFP, or HER2CAR-NIS T cells on Days 7, 10, and 13. Tumor burden was assessed biweekly via BLI. PET imaging was performed on Days 17 and 28. HER2CAR-GFP mice were sacrificed for tissue analysis before reaching endpoint and excluded from survival analysis. Endpoint criteria included tumor radiance ≥8 × 10⁸ p/s/cm²/sr, ascites, hemorrhage, labored breathing, or ≥20% weight loss. Euthanasia was performed via isoflurane overdose.

### Imaging procedures

*In vivo Bioluminescence Imaging (BLI):* Mice were anesthetized with 1-2% isoflurane and imaged using an IVIS Lumina XRMS system (Revvity) following IP injection of BLI substrates: Akalumine-HCl (Sigma-Aldrich, Cat#808350-5MG) (5 mM, 100 μL), D-luciferin (Syd Labs, Cat#MB000102-R70170) (150 µg/mL, 100 μL), Nano-Glo FFZ (Promega, Cat#N4100) (2.3 mM, 50 μL) to track AkaLuc-, FLuc- or Antares-expressing cells, respectively. Images were acquired every 2 min for up to 30 min or until peak radiance was reached. Dual AkaLuc (CAR-T) and Antares (cancer cells) imaging was spaced 1-2 days apart to avoid residual bioluminescence overlap. Data were analyzed using Living Image and Aura Imaging Software (v4.0.7).

*[^18^F]-TFB PET Imaging:* Mice were anesthetized with 2% isoflurane and injected via the tail vein with 10.71 ± 0.68 MBq of [^18^F]-TFB in 50-150 μL of sterile saline. Static PET scans were acquired for 15 min, beginning 30 min post-injection, using a Siemens Inveon® small animal PET system (Siemens Medical Solutions Inc., USA). Throughout imaging, animal breathing rate and body temperature were monitored and maintained between 40-70 bpm and at 37 °C, respectively. Whole-body images were reconstructed using a three-dimensional ordered subsets expectation-maximization (3D-OSEM) algorithm. PET signal analysis was performed by manual segmentation of volumes of interest (VOIs) using Horos Project software (v4.0.0.RC5), and standardized uptake values (SUV) were measured in VOIs corresponding to muscle (left hind limb), subcutaneous tumors, and thoracic and abdominal cavities. Organs with known physiological uptake or involved in radiotracer excretion (e.g., thyroid, stomach, kidneys, bladder) were excluded from analysis. SUV was calculated with Equation 3:


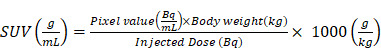

(Eq. 3)

Contrast-to-noise ratio (CNR) was calculated with equation 4:


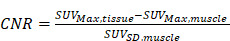

(Eq. 4)

### Histology and Immunofluorescence Staining

Organ tissues and tumors were collected after sacrifice and were fixed overnight in 4% paraformaldehyde (PFA) in PBS at 4 °C. Following tissue processing, paraffine-embedding and H&E staining were performed at the Molecular Pathology Core Facility at Robarts Research Institute, Western University. For immunofluorescence staining, 4% PFA-fixed specimens were sequentially incubated overnight in 10%, 20%, and 30% sucrose solutions (in 1X PBS) before OCT embedding and cryo-sectioning. 10 mm-thick frozen sections were thawed and permeabilized with 0.25% (v/v) Triton X-100 (Sigma) in PBS for 10 min and blocked for 1 h with 5 % normal goat serum, and 1 % BSA in PBS at RT. Next, tissue sections were incubated overnight at 4 °C with rabbit recombinant anti-human CD3 epsilon antibody [EP449E] (Abcam, Cat# ab52959, Cambridge, MA, USA) at 1:150 dilution, 1.5 μg/mL final concentration. After three consecutive washes with PBS, 5 min each, a goat anti-Rabbit IgG (H+L) Cross-Adsorbed Alexa Fluor™ 647 secondary antibody (Invitrogen, Cat# A-21244, Eugene, OR, USA) (1:400 dilution; 3.2 μg/ml final) was applied for 1h at RT. Sections were counterstained with Mounting Medium containing DAPI (Abcam, Cat# ab104139, Cambridge, MA, USA). Slides were imaged with 4x/NA 0.13 PhL or 10x/NA 0.30 Ph1 Olympus UPlanFL N objectives in a Revolve-2 Microscope (ECHO, San Diego, CA).

### Statistics

Unless otherwise stated, data are presented as mean ± standard deviation (SD) from at least three independent experiments or animals. Statistical significance was defined as p < 0.05. Normality of data distribution was assessed using the Shapiro-Wilk test. For longitudinal and repeated measures analyses, either a mixed-effects model or repeated-measures ANOVA was used, depending on variance and distribution characteristics. When appropriate, data were log-transformed to meet model assumptions. For non-normally distributed data, multiple group comparisons were performed using multiple Mann-Whitney tests. The Greenhouse-Geisser correction was applied to account for violations of sphericity. Multiple pairwise comparisons were adjusted using the Holm-Šidák method, and when comparing to a designated reference group, the Dunnett's method was used. Survival analyses were performed using the log-rank (Mantel-Cox) test. All statistical analyses were conducted using GraphPad Prism (version 10.0.0 (131) for macOS; GraphPad Software, La Jolla, CA, USA; www.graphpad.com).

## Results

### Efficient TRAC-targeted CRISPR-Cas9 editing of primary human T cells with a CAR and imaging reporter genes

**Figure 1:**
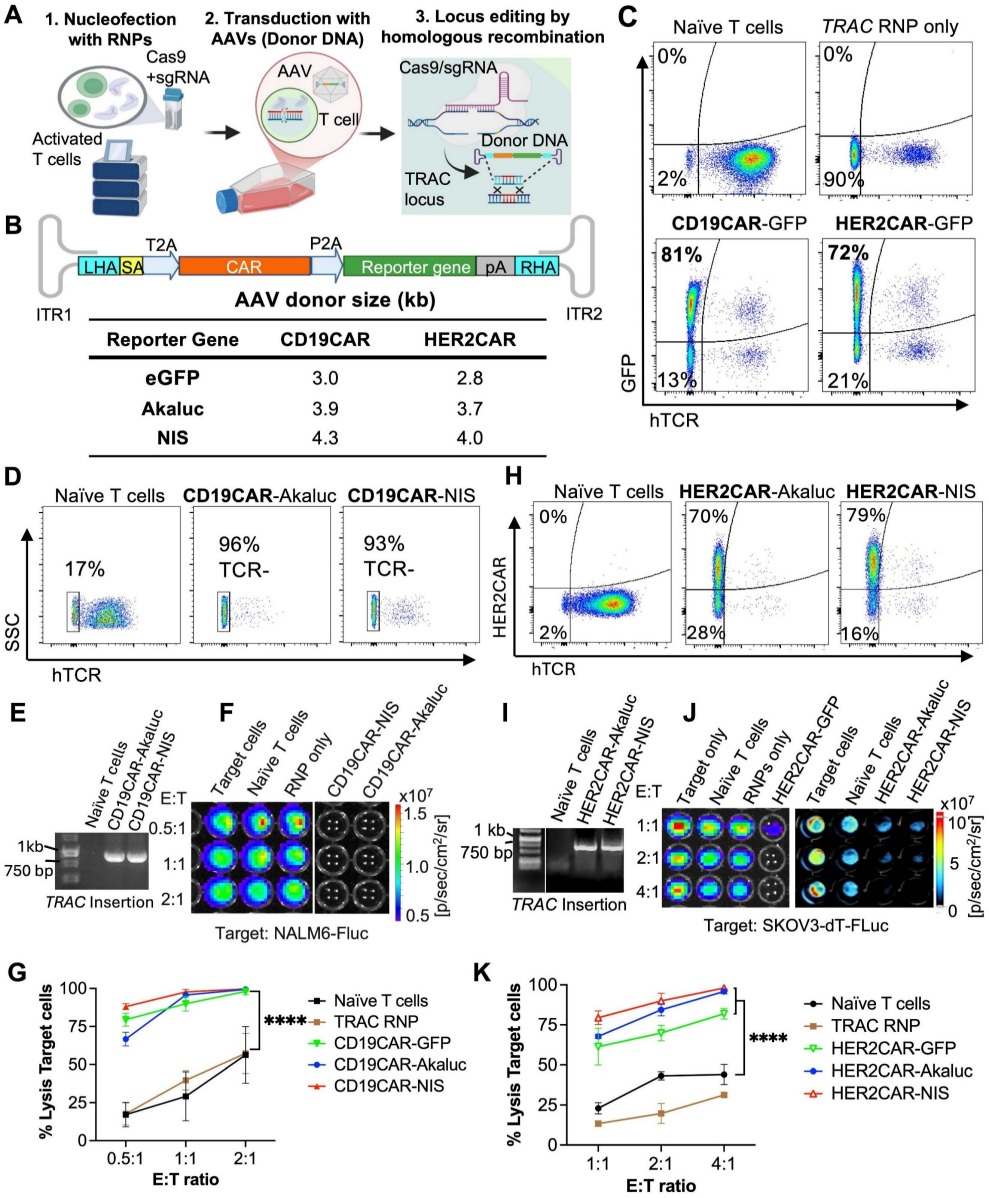
** Efficient genome editing of primary human T cells with CAR and imaging reporter genes. A)** Genome editing workflow using CRISPR-Cas9 technology and adeno-associated viruses (AAV) to engineer human T cells at the *TRAC* locus with CAR and imaging reporter genes for fluorescence (eGFP), bioluminescence (AkaLuc), or PET (NIS). Created in BioRender. Ronald, J. (2022) https://BioRender.com/w24n349. **B)** Schematic of generic donor construct cloned within AAV inverted terminal repeats (ITR). LHA and RHA: left and right homologous recombination arms; SA: splice acceptor site; T2A and P2A: self-cleaving peptide sequences; CAR: chimeric antigen receptor; pA: poly A tail. The table shows the full size of each DNA donor. **C, D, H)** Flow cytometry of primary T cells labeled with an antibody to the human T cell receptor (hTCR), GFP fluorescence, or a fluorokine against the CAR, showing *TRAC* locus editing efficiency.** E, I)** Junction PCR confirming successful CRISPR/Cas9-mediated AAV donor insertion into the *TRAC* locus.** F, G, J, K)** BLI-based cytotoxicity assays showing targeted CD19-CAR and HER2-CAR killing at various effector (E; T cell) to target (T; NALM6-Fluc or SKOV3-dT-Fluc cells) ratios, 24 h after co-culture. Two-way ANOVA followed by Holm-Šidák's multiple comparisons test. Significance is shown only at the highest E:T ratio: ****p < 0.0001. Data are shown as mean ± SD from three independent experiments (N = 3).

To create tRACE-CAR T cells based on the work of Eyquem *et al.* 21, we developed adeno-associated virus serotype 6 (AAV6) donor vectors co-expressing a CAR and an imaging reporter gene with homologous arms (HA) targeting the first exon of the *TRAC* locus to disrupt TCRa expression (**Figure 1A**). These AAV6 vectors contained a promoter-less CD19CAR or HER2CAR transgene positioned after a splice-acceptor sequence and a T2A self-cleaving sequence to drive expression from the endogenous *TRAC* promoter (**Figure 1B**). For imaging, the cDNA of eGFP, AkaLuciferase (AkaLuc) for BLI 49 or human sodium iodide symporter (NIS) for PET 50 was cloned after the CAR. Given HA length can influence homology-directed repair efficiency 51, we tested vectors with 200-bp and 600-bp HA lengths and varying AAV6 doses (1x10^5^-1x10^6^ viral genomes (VG) per cell) following nucleofection of activated human T cells with *TRAC*-targeted Cas9-gRNA ribonucleoproteins (**Figure S1A**). High-efficiency editing (>85% GFP+TCR-) was achieved even at the lowest AAV dose, and no notable differences were found between AAVs with different HA lengths (**Figure S1B**). Overall, comparable results were obtained when switching either the CAR or reporter gene, achieving >90% efficiency in knocking out TCR and rendering 70-80% CAR+TCR- cells (**Figure 1C-D, H**). For the CAR-GFP constructs, CAR expression correlated with GFP expression, and CAR-GFP expression did not change the proportions of CD4+ and CD8+ T cell subtypes compared to the parental cells from the same donor (**Figure S1C-E**). AAV6 donor integration at the *TRAC* locus was confirmed by junction PCR from genomic DNA (**Figure 1E-I**).

Cytotoxicity assays of CD19-positive leukemia (**Figure 1F-G**) or HER-positive ovarian cancer cells (**Figure 1J-K**) expressing Firefly luciferase showed that T cells engineered with either CAR construct performed well *in vitro*, effectively killing cancer cells regardless of the reporter gene co-expressed. As expected, the number of CAR-AkaLuc T cells correlated with the BLI signal and uptake of the NIS-targeted radiotracer ^18^F-tetrafluoroborate (^18^F-TFB) 52 was 4.89-fold higher in *TRAC*-edited CD19CAR-NIS T cells compared to naïve T cells (**Figure S2**).

### Immunophenotypic characterization during CAR-T cell expansion

**Figure 2:**
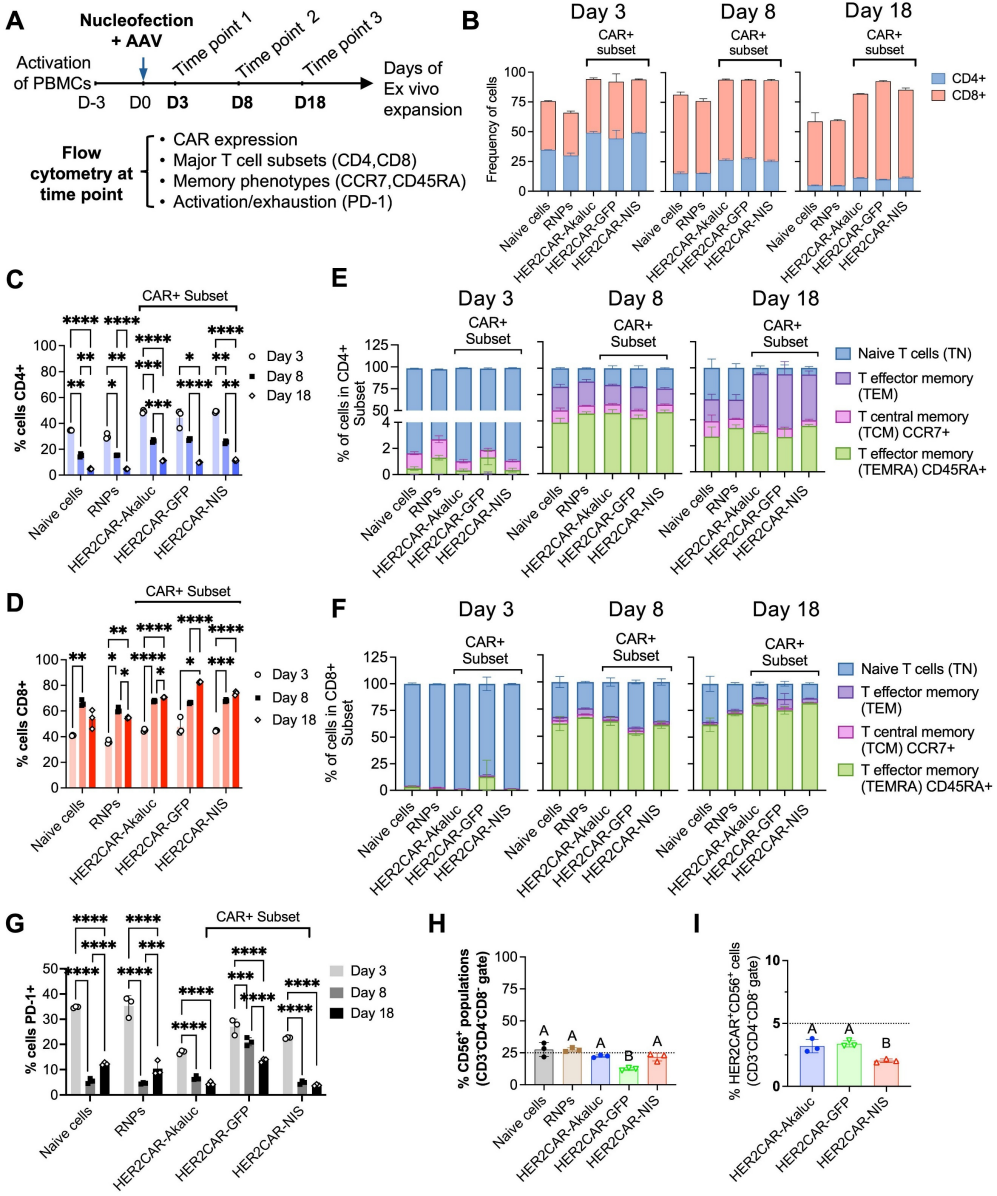
**Phenotypic characterization of engineered CAR-T cells derived from hPBMCs over time. A)** Experimental workflow outlining activation, nucleofection, and longitudinal evaluation of hPBMCs engineered to express HER2-specific CAR constructs. Cells were activated with anti-CD3/CD28 beads, nucleofected with AAV vectors, and expanded *ex vivo* with hIL-2. Flow cytometry analysis was performed at days 3, 8, and 18 post-nucleofection. **B-D)** Quantification of CD4⁺ and CD8⁺ T cell subsets across time points and conditions, including naïve (mock), RNPs (no AAV donor), HER2CAR-Akaluc, HER2CAR-GFP, and HER2CAR-NIS. **E-F)** Distribution of memory subsets—naïve T cells (TN), central memory (TCM), effector memory (TEM), and terminally differentiated effector memory T cells re-expressing CD45RA (TEMRA)—within CD4⁺ and CD8⁺ CAR⁺ populations, showing dynamic phenotypic shifts over time. **G)** PD-1 expression levels on total viable and CAR+ across conditions and time points, indicating similar trends in T cell exhaustion among groups after 18 days *ex vivo* expansion. **H-I)** CD56 expression in bulk cell populations at day 18 post-nucleofection, providing insight into potential NK cell contaminants (CD3^-^CD4^-^CD8^-^) in the engineered PBMC preparations. Different letters above bars indicate statistically significant differences (p < 0.05) between groups; groups sharing the same letter are not significantly different. Data were analyzed using two-way or one-way ANOVA followed by Tukey's multiple comparison test. Asterisks denote statistical significance as follows: p < 0.05 (*), p < 0.01 (**), p < 0.001 (***), and p < 0.0001 (****).

To assess the impact of *ex vivo* expansion and reporter gene expression on engineered HER2CAR-T cells, we performed immunophenotyping flow cytometry at days 3, 8, and 18 post-nucleofection (**Figure 2**). Across all conditions, a consistently higher proportion of HER2CAR⁺ CD8⁺ T cells relative to CD4⁺ T cells was observed, regardless of PBMC engineering status (**Figure 2B-D**). This is consistent with prior reports showing IL-2 preferentially expands CD8⁺ T cells due to elevated IL-2 receptor expression and distinct signaling dynamics 53. Phenotypic analysis revealed a decrease in PD-1 expression and an increase in CCR7⁻CD45RA⁺ cells, primarily within the CD8⁺ subset, indicative of terminally differentiated memory T cells (**Figure 2E-G**).

As the HER2CAR-T cells were engineered directly from PBMCs, we next evaluated potential NK cell carryover. At day 18 post-nucleofection, a relatively low percentage (<25%) of CD56⁺ NK cells (CD3⁻CD4⁻CD8⁻) persisted, of which less than 5% were CAR-positive (**Figure 2H-I**). Overall, the immune composition remained relatively unchanged across all conditions, suggesting that co-expression of CAR and the different reporter genes did not overly alter the immune composition of the final CAR-T cell therapy product.

### *In vivo* antitumor efficacy and bioluminescence imaging of tRACE-CAR T cells in leukemia and ovarian models

**Figure 3:**
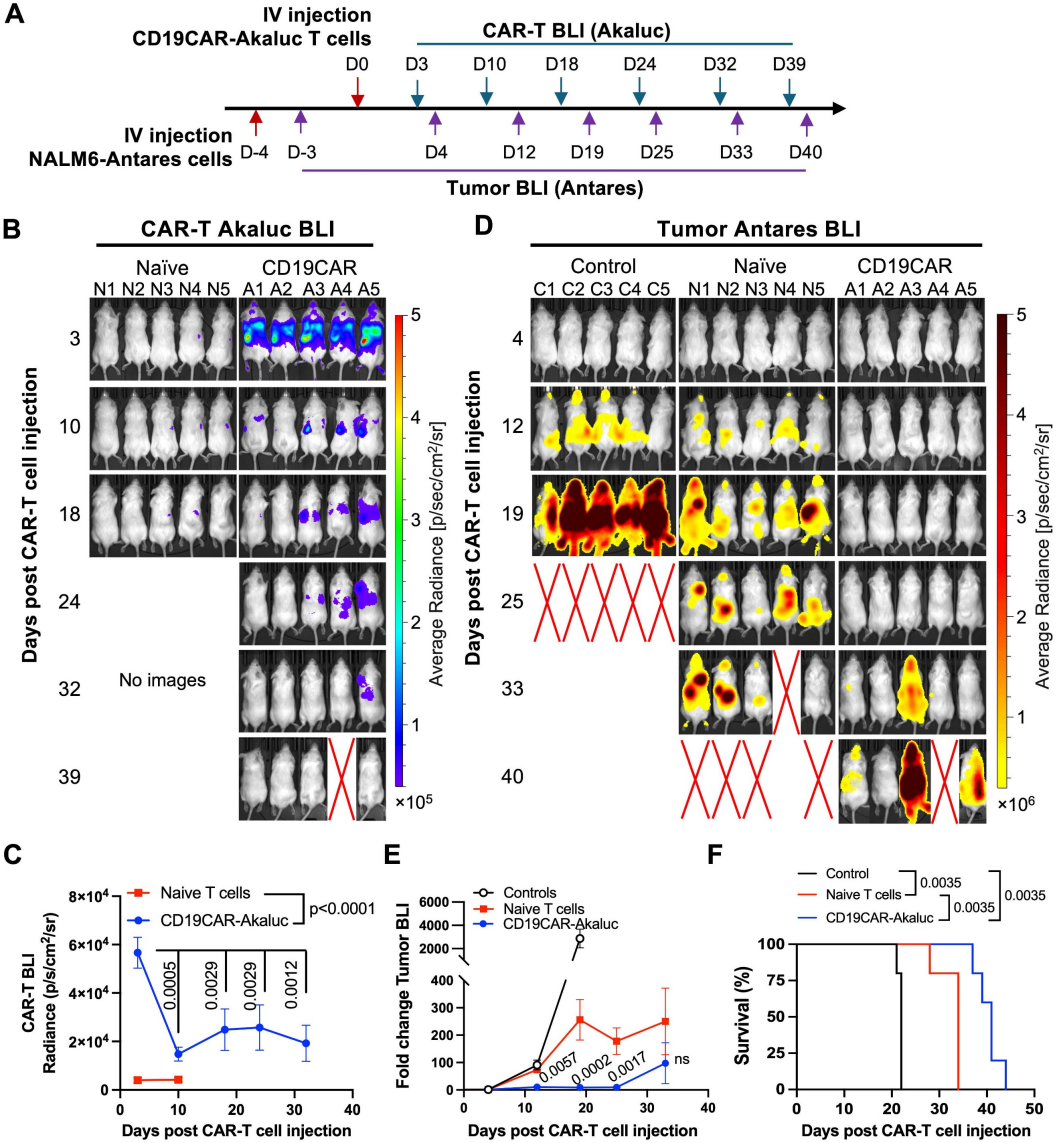
***In vivo* tracking of human CD19CAR-AkaLuc T cells in a mouse model of NALM6 leukemia. A)** Experimental timeline of the intravenous (IV) NALM6-Antares leukemia mouse model treated with CD19CAR-AkaLuc T cells (IV administration).** B)** CD19CAR-AkaLuc BLI at various time points, starting 3 days (D3) post CAR-T cell injection. Naïve images included up until day 18 to assess the background Akalumine signal. **C)** Quantification of whole-body AkaLuc BLI signal. Comparisons between CD19CAR-AkaLuc and naïve T cells on days 3 and 10 were performed using two-way ANOVA. Longitudinal analysis employed a mixed-effects model with Geisser-Greenhouse correction and Holm-Šidák's post hoc test. **D)** BLI of NALM6-Antares tumor in control (no treatment), naïve T cell, and CD19CAR-AkaLuc T cell-treated mice. Red X denotes animal death prior to imaging. Baseline imaging (Day 0) did not show tumor signal above background; therefore, the earliest time point is shown. **E)** Quantification of tumor burden by Antares BLI. Mixed-effects model with Geisser-Greenhouse correction and Šidák's post hoc test. **F)** Kaplan-Meier survival curve. Survival analysis performed using Log-rank (Mantel-Cox) test. Data are shown as mean ± SD; N = 5 mice per group. Statistical significance indicated; ns, not significant.

To ensure our engineered tRACE-CAR T cells were functional *in vivo*, our first study included mice with Firefly luciferase (Fluc)-expressing NALM6 as a systemic disease model that were treated intravenously (IV) with 3x10^6^ CD19CAR-GFP T cells (**Figure S3A**). Compared to naïve T cell treatment, CD19CAR-GFP infusion significantly reduced tumor BLI by day 6 and improved survival (**Figure S3B-D**). We then used a dual BLI system to track both NALM6 cancer cells (expressing Antares) and CAR-T cells (expressing AkaLuc) in individual mice. Mice were injected IV with NALM6 cells followed by an IV dose of 10x10^6^ CD19CAR-T cells or naïve T cells 4 days later (**Figure 3A**). AkaLuc CAR-T signal, which was mainly localized to the thoracic region and sparsely in the abdomen (**Figure 3B**), decreased 3.8-fold from Day 3 to Day 10 but remained stable thereafter (**Figure 3C**). Notably, Antares cancer cell signal (**Figure 3D**) was reduced from Days 12 to 25 post-treatment in the CAR-T group compared to naïve T cell and untreated control mice, which resulted in significantly extended survival (**Figure 3E-F**).

**Figure 4:**
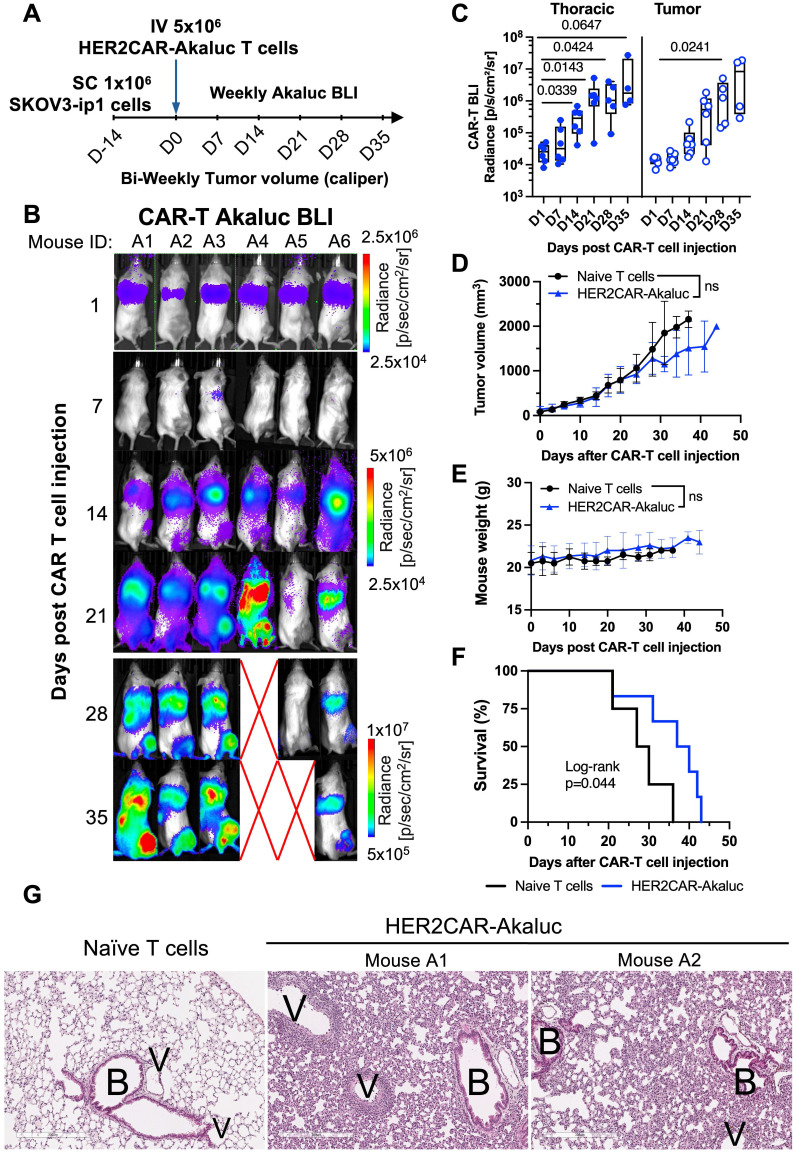
** BLI tracking of HER2CAR-AkaLuc T cell dynamics in a subcutaneous model of ovarian cancer. A)** Timeline of the subcutaneous SKOV3-ip1 ovarian cancer model for longitudinal monitoring of HER2CAR-AkaLuc T cell localization and expansion. **B)** BLI of HER2CAR-AkaLuc-treated mice post IV CAR-T cell injection. Color scale represents average radiance (p/sec/cm²/sr). Red X denotes animal death prior imaging. **C)** Boxplot quantification of HER2CAR-AkaLuc signal in thoracic and tumor regions of interest (ROIs). Data compared to baseline (Day 0) using a mixed-effects model with Geisser-Greenhouse correction and Dunnett's multiple comparisons test. Boxes represent interquartile range (IQR) and median; whiskers denote minimum and maximum values.** D, E)** Tumor volume and mouse body weight monitoring, respectively. Both datasets analyzed using two-way ANOVA followed by Holm-Šidák's multiple comparisons test. **F)** Kaplan-Meier survival analysis of naïve T cell (n = 4) vs. HER2CAR-AkaLuc (n = 6) treated mice, Log-rank (Mantel-Cox) test. **G)** Histology of lung tissues at endpoint. H&E-stained sections from naïve T cell-treated and HER2CAR-AkaLuc-treated mice. B, bronchioles; V, vasculature. Scale bar = 300 μm. Data are presented as mean ± SD unless otherwise indicated. Statistical significance indicated; ns, not significant.

We then evaluated tracking of AkaLuc-expressing HER2CAR-T cells in mice bearing HER2-positive ovarian SKOV3-ip1 subcutaneous (SC) xenografts (**Figure 4A**). One day following IV infusion of 5x10^6^ HER2CAR-T cells, AkaLuc signal was primarily concentrated in the thoracic region (**Figure 4B**). By Day 14, HER2CAR-T cells localized to SC tumors, and expanded over time with a significant increase in AkaLuc CAR-T signal detected in the lungs (**Figure 4C**). Near endpoint, mice receiving CAR-T cells showed a trend towards lower tumor volume compared to the naïve T cell group, without changes in body weight between treatment groups (**Figure 4D-E**). Based on tumor volume and other humane endpoints (e.g., tumor ulceration) set in our experimental protocol, HER2CAR-T cell treatment significantly improved mouse survival compared to naïve T cell**s (Figure 4F**). Histological comparison of lung tissues at endpoint revealed that, in contrast to naïve T cell-treated mice, HER2CAR-AkaLuc treatment induced extensive lung remodeling, including alveolar narrowing, septal thickening, and perivascular inflammation (**Figure 4G**). Overall, our results show that our tRACE-CAR system allowed for effective BLI detection of therapeutic cells expressing different CARs in both hematological and solid pre-clinical cancer models.

### [^18^F]-TFB PET tracking of HER2-targeted tRACE-CAR cells in an ovarian cancer subcutaneous xenograft model

**Figure 5:**
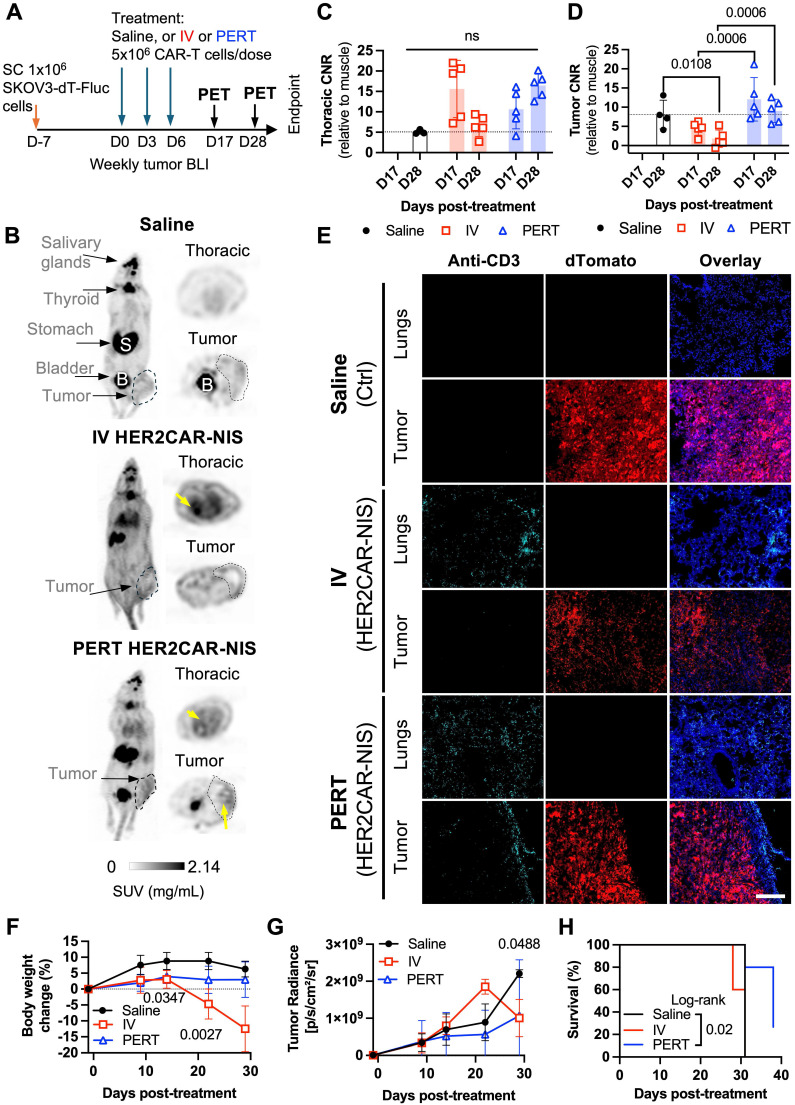
** PET imaging and tissue-level characterization of HER2CAR-NIS T cells in a subcutaneous ovarian cancer model. A)** Experimental timeline of SC inoculation of SKOV3-ip1 (dTomato^+^Fluc^+^) tumor cells followed by three doses of IV or peritumoral (PERT) HER2CAR-NIS T cells.** B)** PET maximum intensity projections (MIP) showing coronal and axial views of representative mice on Day 28. Grayscale bar indicates SUV (g/ml). Yellow arrows highlight increased radiotracer uptake. Physiological uptake shown in salivary glands, thyroid, stomach, and bladder. Tumor locations outlined with dashed circles. **C, D)** Contrast-to-noise ratio (CNR) values relative to muscle from thoracic and tumor volumes of interest (VOIs). Data analyzed using two-way ANOVA with Holm-Šidák's multiple comparisons test. Dotted line represents average CNR of saline-treated controls. Symbols indicate individual animals.** E)** Immunofluorescence of CD3+ cells (cyan) in lung and tumor sections. Tumor cells express dTomato (red); nuclei stained with DAPI (blue). Scale bar = 200 µm. **F)** Percent body weight change post CAR-T cell treatment analyzed with a mixed-effects model with Holm-Šidák correction; significant p-values shown for IV vs. Saline comparisons. **G)** Tumor radiance (Fluc BLI) over time analyzed with mixed-effects model with Holm-Šidák correction. **H)** Kaplan-Meier survival curves analyzed using Log-rank (Mantel-Cox) test. Data are shown as mean ± SD; N ≥ 4 mice per group. Statistical significance indicated; ns, not significant.

Based on our HER2CAR BLI data showing both tumor and lung accumulation, we next evaluated the ability to track on- and off-tumor localization of tRACE-CAR T cells using the sodium iodide symporter (NIS), a clinically translatable, human-derived PET reporter. Mice bearing subcutaneous SKOV3-ip1 tumors expressing FLuc were randomized to receive IV injections of 5×10⁶ HER2CAR-NIS T cells or saline (**Figure 5A**). To potentially enhance therapeutic efficacy, an additional cohort received peritumoral (PERT) injections of CAR T cells, as previously described 54. [¹⁸F]-tetrafluoroborate ([¹⁸F]-TFB) PET imaging was performed on Days 17 and 28 post-treatment (Day 28 only for saline controls), and tumor BLI was conducted weekly.

Consistent with the known biodistribution of [¹⁸F]-TFB 55, saline-treated mice exhibited radiotracer uptake in the thyroid, salivary glands, stomach, and bladder (**Figure 5B**). In contrast, mice treated with HER2CAR-NIS T cells via either IV or PERT injection displayed pronounced radiotracer accumulation in the lungs which was absent in saline-treated controls. Quantitative analysis revealed lung-to-muscle contrast-to-noise ratio (CNR) PET values exceeding 5, the Rose criterion for detectability 56, for most PERT-treated mice on Days 17 and 28, and for IV-treated mice on day 17, but not Day 28 (**Figure 5C**). By day 28, IV-treated mice exhibited signs of morbidity, including significant weight loss (**Figure 5F**), labored breathing, lack of responsiveness, dehydration, and a low body score. Many IV-treated mice also lacked detectable bladder signal, suggesting impaired renal clearance of the radiotracer (**Figure 5B**). Although PET signal (SUVMax) in the lungs remained elevated in these mice (**Figure S4A**), increased signal in muscle (**Figure S4B**) led to reduced lung-to-muscle CNR at Day 28. Notably, PERT-treated mice showed delayed pulmonary accumulation of HER2CAR-NIS T cells, with significantly lower lung signal at day 17 compared to IV-treated animals that then increased by day 28 (**Figure S4A**). Immunofluorescence staining of lung sections from IV- and PERT-treated mice that exhibited high pulmonary PET signal confirmed the presence of human CD3⁺ T cells at endpoint, supporting the imaging findings (**Figures 5E and Figure S4D**, lungs panel).

Tumor-to-muscle CNR PET values were comparable between IV-treated and control mice (**Figure 5D**), consistent with the absence of human CD3⁺ T cells in tumor sections from these groups (**Figure 5E**, tumor panel), and a lack of tumor response (**Figure 5G**). In contrast, PERT-treated mice exhibited significantly higher CNR values compared to the IV-treated group at both Days 17 and 28. Correspondingly, CD3⁺ T cells were observed at the tumor periphery in PERT-treated animals (**Figures 5E and Figure S4D**, tumor panel). While tumor BLI signal did not differ significantly between groups (**Figure 5G**), 2 out of 5 PERT-treated mice demonstrated a reduction in tumor burden via BLI (**Figure S4E-F**), which was associated with significantly improved survival compared to saline-treated control mice (**Figure 5H**).

### PET tracking of tRACE-CAR T cells following locoregional administration in an intraperitoneal ovarian cancer model

Although subcutaneous tumor models are informative for early imaging studies, ovarian cancer predominantly disseminates within the intraperitoneal (IP) cavity 57. To better mimic the clinical context, we next assessed reporter imaging in an experimental IP metastasis model of ovarian cancer. To attempt to minimize CAR T cell entrapment in the lungs, we administered tRACE-CAR T cells via IP injection. Locoregional delivery of CAR T cells has demonstrated therapeutic potential in several tumor types, including ovarian cancer 58-60.

**Figure 6:**
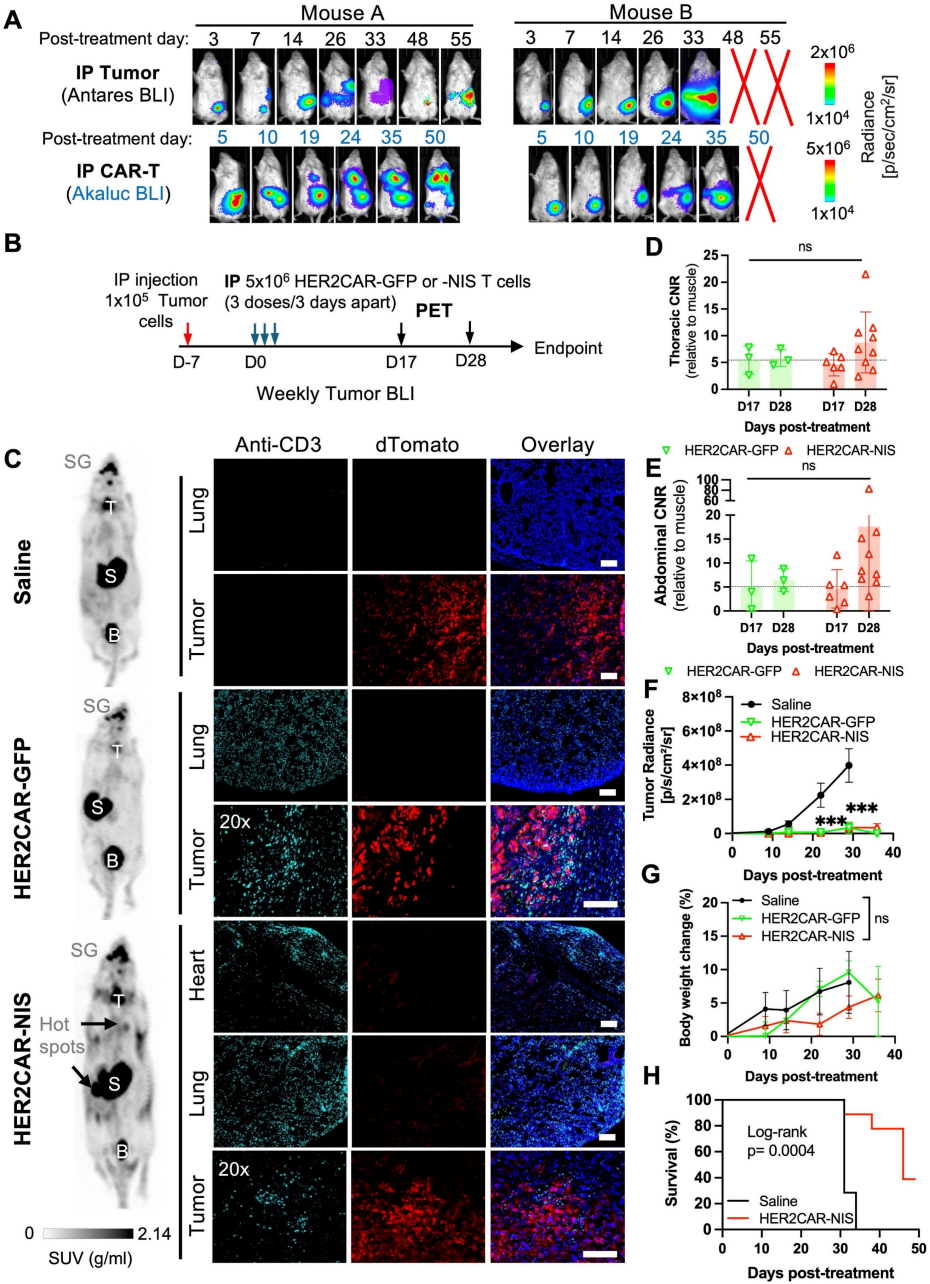
**Imaging of HER2CAR T cells after locoregional administration in an orthotopic model of ovarian cancer. A)** Dual BLI tracking of IP-injected HER2CAR-AkaLuc T cells and SKOV3-ip1-Antares tumor cells in the same mouse. **B)** Timeline of IP SKOV3-ip1(dT+Fluc+) cancer model with IP-administered HER2CAR-NIS T cells. D17 and D28 indicate PET imaging time points post first CAR-T dose. **C)** Representative ^18^F-TFB PET MIP images at D28 for mice treated with saline, HER2CAR-GFP, or HER2CAR-NIS T cells. Black arrows indicate ^18^F-TFB hotspots; physiological uptake shown in thyroid (T), salivary glands (SG), stomach (S), and bladder (B). Right: anti-CD3 immunofluorescence (cyan) of matched tissues; tumor cells express dTomato (red), nuclei stained with DAPI (blue). Scale bars = 100 µm. **D, E)** CNR values (relative to muscle) from thoracic and abdominal VOIs; data were analyzed using a non-parametric Mann-Whitney test with Holm-Šidák correction for multiple comparisons. **F)** Tumor radiance per group. **G)** Percent body weight change post CAR-T treatment; mixed-effects model with Holm-Šidák correction. **H)** Kaplan-Meier survival comparison between Saline and HER2CAR-NIS groups analyzed using Log-rank test. PET imaging group sizes: Saline (N = 4), HER2CAR-GFP (N = 3), HER2CAR-NIS (N = 9). Data are shown as mean ± SD. Asterisks denote statistical significance as follows: p < 0.001 (***); ns, not significant.

To determine optimal time points for PET imaging in this model, we injected CRISPR-edited HER2CAR T cells expressing AkaLuc into two mice bearing IP SKOV3-ip1 tumors engineered to express Antares. We then performed dual BLI over time (**Figure 6A**). Qualitatively, Mouse A responded to therapy, based on minimal cancer BLI signal increase over time, while Mouse B did not. Both mice consistently showed CAR T cell signals in the abdominal region at all observed time points while Mouse A additionally exhibited an increase in the thoracic region from Days 19 to 50. Based on these dynamics, we selected Days 17 (prior to the appearance of thoracic signal) and 28 (when strong abdominal signals were observed in both mice) as the PET imaging time points.

For our PET imaging study, mice received three IP injections of either saline (n = 7), HER2CAR-GFP T cells (n = 4), or HER2CAR-NIS T cells (n = 9), with a total dose of 15 × 10⁶ cells per mouse (**Figure 6B**). [¹⁸F]-TFB PET imaging showed similar tracer biodistribution in HER2CAR-GFP and saline-treated mice, whereas some HER2CAR-NIS-treated mice exhibited localized tracer accumulation in the lungs (**Figure 6C**). Quantification of [¹⁸F]-TFB uptake based on SUVMax in the thoracic region showed comparable background uptake in muscle tissue and overall thoracic signal across groups (**Figure S5C-D**). However, thoracic CNR values, relative to muscle, were approximately 1.5-fold higher in HER2CAR-NIS mice compared to the HER2CAR-GFP group on Day 28 and 4/10 mice had a CNR of 10 or above (**Figure 6D**). Importantly, immunostaining confirmed CD3⁺ T cells in the lungs of both treated cohorts and in the pericardial tissue of HER2CAR-NIS mice, supporting that the elevated PET signal observed in the HER2CAR-NIS group is attributable to NIS-dependent [¹⁸F]-TFB uptake **(Figure 6C)**. Although we did not have a direct fluorescence label for NIS, we detected strong GFP fluorescence with overlapping CD3 expression, in both the lungs and hearts of HER2CAR-GFP treated mice, indicating that CD3 labelling was predominantly associated with CAR-T cells and not naïve T cells (**Figure S5A-B**).

Some HER2CAR-NIS-treated mice also exhibited focal hot spots of elevated PET signal within the abdomen (**Figure 6C**), as reflected by increased CNR values on Day 28 (**Figure 6E**). CD3⁺ T cells were detected by immunostaining within dTomato⁺ tumors in both HER2CAR-GFP and HER2CAR-NIS cohorts, further supporting the specificity of NIS-mediated PET imaging (**Figure 6C and Figure S5A**). BLI revealed tumor signals confined to the abdominal cavity in all mice (**Figure S5F**). Both HER2CAR-GFP and HER2CAR-NIS groups demonstrated significantly reduced tumor burden compared to saline-treated controls, with no significant differences in treatment efficacy and similar body weight change between GFP- or NIS-expressing cohorts (**Figure 6F-G**). HER2CAR-GFP mice were euthanized after the final PET timepoint for tissue analysis, whereas HER2CAR-NIS-treated mice were monitored for long-term outcomes and exhibited significantly prolonged survival relative to the saline group (**Figure 5H**).

In summary, the tRACE-CAR platform enabled efficient genome editing and multimodal imaging of CAR-T cells, supporting functional tracking in hematologic and solid tumor models. Overall, these findings establish tRACE-CAR as a versatile and informative tool for evaluation of CRISPR-edited cell therapies.

## Discussion

CAR-T cell therapy has revolutionized the treatment of hematologic malignancies and is increasingly being investigated for solid tumors. Nevertheless, heterogeneous clinical responses, on-target/off-tumor toxicities and concerns with lentiviral engineering of CAR-T cells continue to limit efficacy and safety. These challenges underscore the need for improved, real-time tools to monitor CAR-T cell trafficking, persistence, and function within the body. Conventional methods such as biopsies or peripheral blood sampling provide limited insight into spatial and temporal dynamics, highlighting the value of imaging-based approaches to better understand CAR-T behavior *in vivo*.

In this work, we developed and validated an imaging-enabled, CRISPR-based CAR-T engineering platform (tRACE-CAR) that enables non-invasive tracking of edited CAR-T cells *in vivo*. Using a CRISPR-Cas9/AAV6 strategy, we site-specifically integrated both CAR and imaging reporter genes into the *TRAC* locus, allowing expression under endogenous regulatory control and improving genomic editing precision compared with conventional viral transduction 12. In addition, editing at the *TRAC* locus has been shown to reduce tonic signaling and exhaustion 21,61. Through multimodal imaging—bioluminescent imaging for preclinical assessment and NIS-PET for clinical translatability—we visualized CAR-T cell dynamics, trafficking, and therapeutic activity in leukemia and ovarian cancer models. Whole-body imaging revealed distinct patterns of persistence, expansion, and tissue distribution influenced by CAR design, tumor type, and delivery route. Collectively, these findings demonstrate the feasibility and translational potential of tRACE-CAR as a platform for integrating functional imaging into CAR-T development and optimization. This approach builds upon earlier targeted reporter gene strategies while extending their application to primary human T cells for immunotherapy research.

Early reporter gene strategies used zinc-finger nucleases to target the adeno-associated virus integration site 1 (AAVS1) safe-harbor locus, enabling multimodal imaging in stem cell-derived systems 62. Recently, Ashmore-Harris *et al.* (2025) demonstrated TALEN-mediated AAVS1-targeted integration of an hNIS-mGFP reporter enabling longitudinal [^18^F]-TFB-PET imaging of gene-edited hiPSC-derived organoids *in vivo* 63. The advent of CRISPR-Cas9 has since improved the versatility and efficiency of targeted knock-ins across diverse cell types 64-66. For example, Lin *et al.* (2024) used CRISPR-Cas9 to insert a constitutive NIS reporter into AAVS1 in iPSC-derived cardiomyocytes and demonstrated long-term engraftment in the hearts of rhesus macaques using [^18^F]-TFB-PET imaging 66. These studies collectively highlight both the feasibility of CRISPR-mediated reporter integration for regenerative cell therapies and the safety of longitudinal NIS-PET imaging.

We previously applied homology-independent targeted integration (HITI)-based CRISPR editing strategies using minicircles to image metastatic cancer cell populations with BLI and MRI *in vivo* 67. However, this editing approach was highly inefficient and relied on single-cell clones, limiting its applicability in primary immune cells. To overcome these limitations, we implemented a bulk-editing strategy by combining Cas9 RNP nucleofection with AAV6 donors in human T cells and consistently achieved >85% editing at *TRAC* and >70% CAR expression. These efficiencies are comparable to recent reports using Cas9 68, Cas12a 69, non-viral donors 70, and advanced enrichment and base-editing platforms to enable multilocus CRISPR edits at *TRAC* and *B2M* loci 71,72. This supports the scalable, clinically relevant production of imageable CAR-T cells. While our findings demonstrate the feasibility, versatility and potentially improved safety of the tRACE-CAR system, future head-to-head comparisons of viral- and CRISPR-engineered CAR-T cells will be valuable to assess differences in efficiency, phenotype, and therapeutic performance.

BLI is a mainstay for indirect cell tracking in preclinical cancer models due to its ease of use, its high sensitivity to detect live cells, ability to image more than one animal at a time and being relatively affordable. Most studies have used BLI to track treatment response, typically tumor clearance, following CAR-T treatment 73-75. However, these studies can overlook CAR cell trafficking to off-tumor organs, which requires extensive post-sacrifice histological analysis. To address these limitations, recent studies, including our own, have applied dual BLI strategies using orthogonal reporter systems 76,77,29,78, next-generation dual-luciferase designs pairing two luciferase proteins with divergent bioluminescent emission wavelengths (e.g., CBG99 & Akaluc) 78 or spectral unmixing approaches that enable the simultaneous imaging of two distinct luciferases using the same substrate 79. This provides the opportunity to look beyond just treatment response and offers spatiotemporal whole-body information of more than one cell type, such as CAR cell trafficking and tumor cell viability. To the best of our knowledge, this study represents the first use of dual BLI to track CRISPR-edited CAR-T cells in mouse models of blood cancer and solid tumors. Our approach revealed both intratumoral accumulation and off-tumor trafficking, including significant lung localization of HER2CAR-T cells in mice with ovarian tumors, even following intraperitoneal injection.

In this study, AkaLuc (BLI) and NIS (PET) reporters were chosen for their superior sensitivity and tissue penetration, whereas GFP was used as control imaging reporter for *in vitro* CAR-T characterization, and *in vivo* experiments, given the limited applicability of fluorescence imaging for deep tissue biodistribution. Targeted integration of the CAR and reporter transgenes at the *TRAC* locus—as verified by loss of TCR expression and junction PCR—enabled sensitive detection of CAR-T cells using BLI and PET, despite the likely low copy number (~1-2 per cell) resulting from CRISPR/AAV-mediated knock-in. Although transgene copy number and off-target integration was not quantified in this study, such analyses will be interesting for future studies to confirm genomic integrity and reduce variability, particularly for clinical translation.

While NIS has background uptake in tissues like thyroid and bladder and modest sensitivity (~10⁴-10⁵ cells), it remains a clinically relevant reporter due to its safety, tracer compatibility, and potential for both imaging and ablation 80. Future strategies could benefit from PET reporters with alternative tracer biodistributions 42 or incorporate radiolabelled antibody- and antigen-based PET imaging strategies 81,82. However, non-genetic PET tracing approaches may yield incomplete labeling, increased background, reduced clearance and dependence on antigen expression. By contrast, integrating a PET reporter like NIS at a specific locus ensures stable, selective CAR/reporter expression and tracer uptake across all modified T cells and daughter cells.

Our findings are consistent with previous NIS-based PET studies tracking CAR-T cells 34-36, including model-dependent persistence in triple negative breast cancer 35 and detection of lung-localized toxicity in a leukemia model 36. Previously, we demonstrated that IP-injected HER2CAR-NIS NK-92 cells remained within the peritoneal space in ovarian tumor-bearing mice without evidence of toxicity 29, whereas here, HER2CAR-NIS T cells showed lung accumulation (as in agreement with our BLI data), reinforcing that trafficking and toxicity depend on both cell type and delivery route. Our imaging data support locoregional delivery to enhance efficacy and reduce systemic toxicity, in line with prior ovarian cancer studies 59,60,83,84, and consistent with known HER2CAR-related lung toxicities in mice and a patient 6,46.

In this work, all *in vivo* experiments were conducted using unpurified engineered PBMC products as this was a feasibility-stage study intended to capture representative trafficking behavior of CAR-T cells in the bulk cell product. The co-expression of imaging reporters with the CARs did not appear to disrupt the overall immune composition or differentiation compared to non-modified naïve T cells and TRAC-edited (RNP) controls, supporting the compatibility of the tRACE-CAR platform with functional CAR-T cell production. Future studies incorporating CAR-positive but reporter-negative T cell controls will help further assess the specific impact of reporter gene insertion on T cell viability, distribution, and therapeutic function. While the *TRAC* locus restricts functional transgene expression to T cells, low-level AAV transduction or transient expression in other PBMC subsets cannot be completely excluded, as observed with a low level of CAR+ NK cells carried over in the unpurified cell therapy product. However, standard immunomagnetic selection protocols could be used to selectively purify specific cell populations for future clinical applications.

The immunophenotypic characterization of engineered CAR-T cells revealed that IL-2-based expansion protocols significantly influence cell composition and differentiation. The predominance of CAR⁺ CD8⁺ T cells and the emergence of a terminal memory phenotype corroborate that IL-2 skewed the final product toward short-lived effector populations 85. While this phenotype supports immediate cytotoxic potential, as seen *in vitro*, it may limit long-term persistence, a factor that could be mitigated by alternative cytokine combinations such as IL-15 or IL-7 86 or reducing long-term exposure to IL-2 by administering the CAR-T cells at lower doses earlier in the culturing timeline. Indeed, the modest PET signal and survival benefit observed following PERT administration of HER2CAR-T cells may be attributed to a reduced expansion capacity or limited *in vivo* persistence but also cannot rule out others causes like suboptimal tumor infiltration and local immunosuppressive factors.

Some of these cellular characteristics may also help explain the observed biodistribution and toxicity profiles. In the case of HER2CAR-T cell lung accumulation, our findings are consistent with Hammill *et al.* (2020), who reported acute pulmonary and cardiac toxicities in mice treated with the same HER2CAR. These effects were attributed to CAR-T cell activation and cytokine release within affected tissues, rather than any cross-reactivity with murine HER2, as no endogenous antigenic target was identified 6. In other scenarios, low-level antigen expression could occur in already inflamed lung epithelium, as described by Haas *et al.* (2023), who observed lung accumulation and toxicity in patients receiving anti-mesothelin CAR-T cells 87. Together with our findings, these reports underscore the importance of investigating mechanisms such as CAR tonic signaling, cytokine-mediated effects, pre-existing inflammation, and tissue-specific tropism that may contribute to CAR-T cell accumulation and toxicity in the lungs. These mechanisms warrant further exploration in preclinical models and could be monitored using our tRACE engineering platform with any CAR-T construct.

Additionally, we observed that mice exhibiting signs of toxicity were those treated with high doses of HER2CAR-T cells administered intravenously. These animals lacked radiotracer signal in the bladder and showed elevated background PET signal, suggesting impaired renal clearance and increased systemic tracer levels. This pattern corresponded with reduced CNR values in PET imaging. Although cytokine release syndrome (CRS) was not directly assessed in this study, the concurrent weight loss and signs of kidney/bladder dysfunction point to systemic inflammation potentially linked to CRS in this model 6. Excessive inflammatory responses, such as those associated with CRS, are known to compromise renal function in patients 88,89, which can impair tracer clearance and contribute to elevated background signal in PET scans.

## Conclusions

In summary, our approach enables precise integration of CAR and imaging reporters into the *TRAC* locus, allowing non-invasive tracking of CAR-T cells *in vivo*. The tRACE-CAR platform supports broader applications, including multiplex editing or universal CAR-T development 90. These findings build on earlier PET imaging work in clinical T cell therapy 40 and open new paths for monitoring CRISPR-edited cell therapies in cancer, infection 91, and autoimmunity 92, with potential for clinical translation.

## Supplementary Material

Supplementary figures.

## Abbreviations

AAV: adeno-associated virus
AAV6: adeno-associated virus serotype 6
AAVS1: adeno-associated virus integration site 1
B2M: beta-2 microglobulin
BLI: bioluminescence imaging
CAR: chimeric antigen receptor
CD: cluster of differentiation
CNR: contrast-to-noise ratio
CRISPR: clustered regularly interspaced short palindromic repeats
CRS: cytokine release syndrome
HA: homologous arm
HER2: human epidermal growth factor receptor 2
HITI: homology-independent targeted integration
HLA: human leukocyte antigen
ICANS: immune effector cell-associated neurotoxicity syndrome
IP: intraperitoneal
IV: intravenous
MRI: magnetic resonance imaging
NALM6: human pre-B acute lymphoblastic leukemia cell line
NIS: sodium iodide symporter
NK: natural killer
NSG: NOD scid gamma
PBMC: peripheral blood mononuclear cell
PCR: polymerase chain reaction
PD-1: Programmed cell death protein 1
PERT: peritumoral
PET: positron emission tomography
ROI: Region of interest
RNP: ribonucleoprotein
SC: subcutaneous
SKOV3: human ovarian carcinoma cell line
SUV: standardized uptake value
T2A: Thosea asigna virus 2A peptide
TALEN: transcription Activator-Like Effector Nuclease
TCR: T cell receptor
[^18^F]-TFB: ^18^F -tetrafluoroborate
TRAC: T cell receptor alpha constant
VOI: volume of interest
