# Supplementary material for: Imaging CRISPR-edited CAR-T cell therapies with optical and positron emission tomography reporters
Source: Theranostics. 2026 Jan 1;16(7):3227–45. doi: 10.7150/thno.119013 (PMC12846014; doi:10.7150/thno.119013)
Supplement: Supplementary file 1 — Supplementary figures. [file thnov16p3227s1.pdf]

Supplementary information

Imaging CRISPR-edited CAR-T cell therapies with optical and positron emission tomography reporters

Rafael Enrique Sanchez-Pupo[1‡*], John Joseph Kelly[1‡], Nourhan Shalaby[1,2], Ying Xia[1], Francisco Manuel Martinez-Santiesteban[1], Jasmine Lau[1], Ivy Elizabeth Verriet[1], Matthew Stefan Fox[2,3,6], Justin Wesley Hicks[2,3], Jonathan Dale Thiessen[2,3,4,5], John Andrew Ronald[1,2,3*]

[1]Robarts Research Institute, University of Western Ontario, London, ON, Canada
[2]Dept. of Medical Biophysics, University of Western Ontario, London, ON, Canada
[3]Lawson Health Research Institute, London, ON, Canada.
[4]Dept. of Medical Physics, CancerCare Manitoba, Winnipeg, MB, Canada
[5]Dept. of Radiology, University of Manitoba, Winnipeg, MB, Canada
[6]Dept. of Physics and Astronomy, University of Western Ontario, London, ON, Canada

‡ These authors are equal contributors to this work and are designated as co-first authors.

*Corresponding authors.

Figure S1

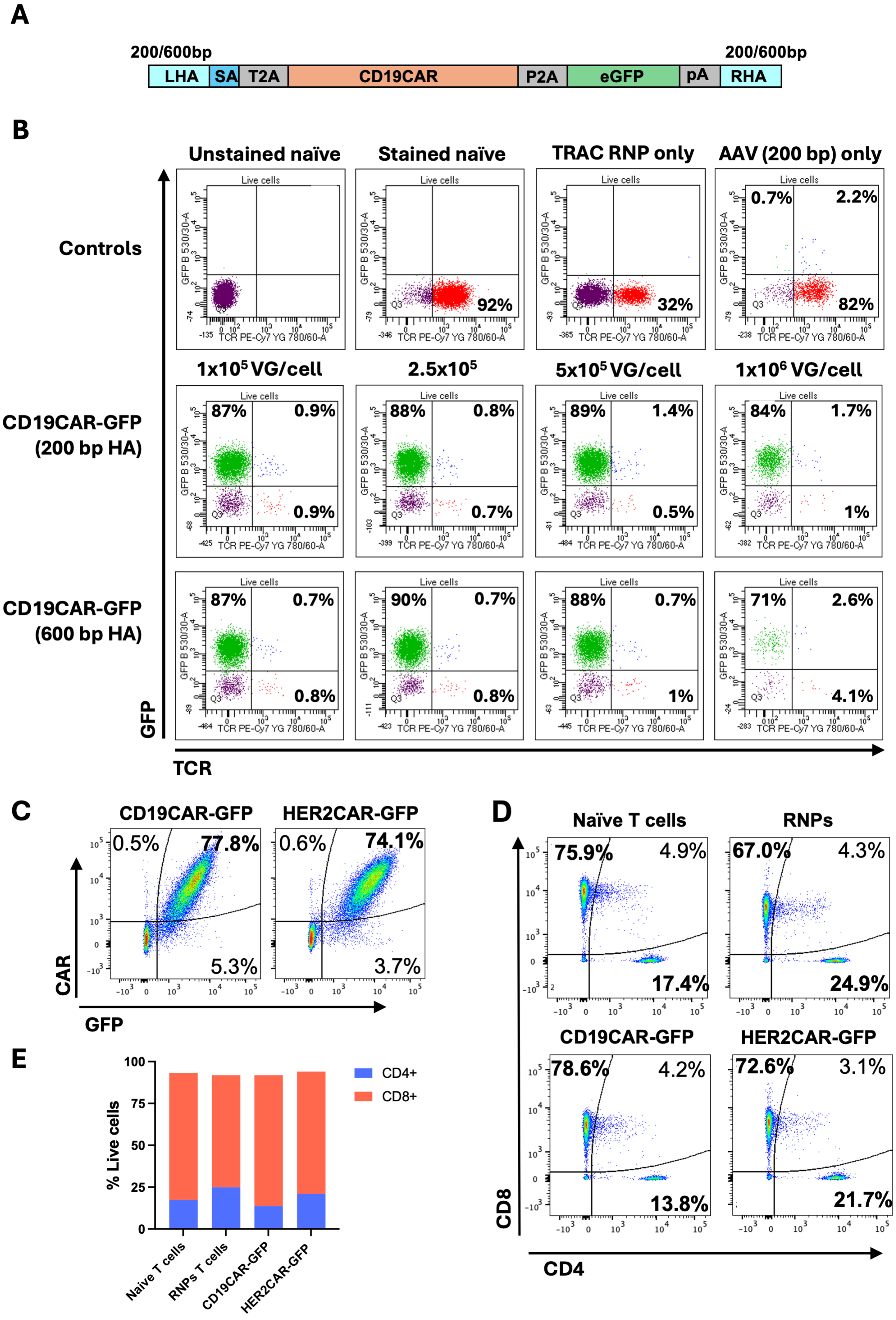

A
200/600bp
200/600bp
LHA
SA
T2A
CD19CAR
P2A
eGFP
pA
RHA
B
Unstained naïve
Stained naïve
TRAC RNP only
AAV (200 bp) only
Controls
92%
32%
0.7%
2.2%
82%
1x10^5 VG/cell
2.5x10^5
5x10^5 VG/cell
1x10^6 VG/cell
CD19CAR-GFP (200 bp HA)
87%
0.9%
0.9%
88%
0.8%
0.7%
89%
1.4%
0.5%
84%
1.7%
1%
CD19CAR-GFP (600 bp HA)
87%
0.7%
0.8%
90%
0.7%
0.8%
88%
0.7%
1%
71%
2.6%
4.1%
Live cells
GFP B 530/30-A
TCR PE-Cy7 YG 780/60-A
GFP
TCR
C
CD19CAR-GFP
HER2CAR-GFP
0.5%
77.8%
5.3%
0.6%
74.1%
3.7%
CAR
GFP
D
Naïve T cells
RNPs
75.9%
4.9%
17.4%
67.0%
4.3%
24.9%
CD19CAR-GFP
HER2CAR-GFP
78.6%
4.2%
13.8%
72.6%
3.1%
21.7%
CD8
CD4
E
% Live cells
CD4+
CD8+
Naive T cells
RNPs T cells
CD19CAR-GFP
HER2CAR-GFP

Figure S1. TRAC-editing efficiency and confirmation of CAR expression in human T cells.

A) Schematic of CD19CAR-GFP donor construct with either 200 bp or 600 bp homologous arms. **B)** Flow cytometry analysis of TRAC locus editing efficiency with CRISPR-Cas9 and AAV6 carrying CD19CAR-GFP donors. Activated human T cells were exposed to various amounts of AAV. 1 x 10^6 AAV genomes/cell caused significant cell death (4th column), hence the lower data outputs. **C)** Flow cytometry analysis of editing efficiency and validation of CAR (labelled with a fluorokine) and GFP co-expression in primary human T cells. **D)** and **E)** Flow cytometry analysis and quantification confirming that CD19CAR- or HER2CAR-GFP knock-in at the TRAC locus does not affect the ratio of CD4+ to CD8+ T cells from the original PBMC donor.

Figure S2.

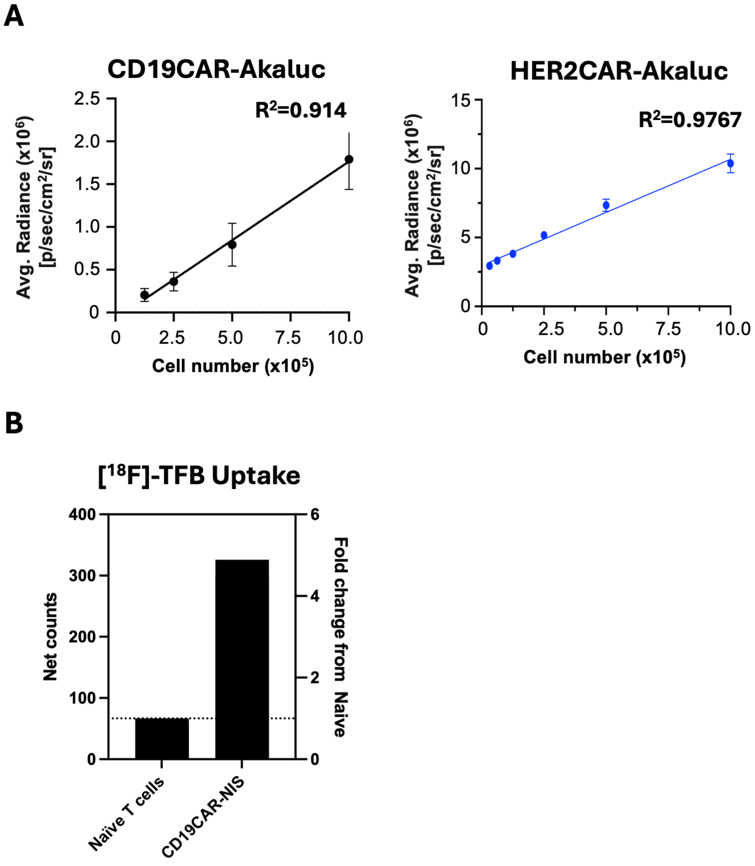

Figure S2. Functional characterization of imaging reporter genes knocked in at the *TRAC* locus.

A) CAR-Akaluc BLI signal correlates with cell number (n = 3). **B)** Gamma counter net counts and fold change from naïve cells of [^{18}F]-TFB uptake into CAR-NIS-expressing T cells.

Figure S3

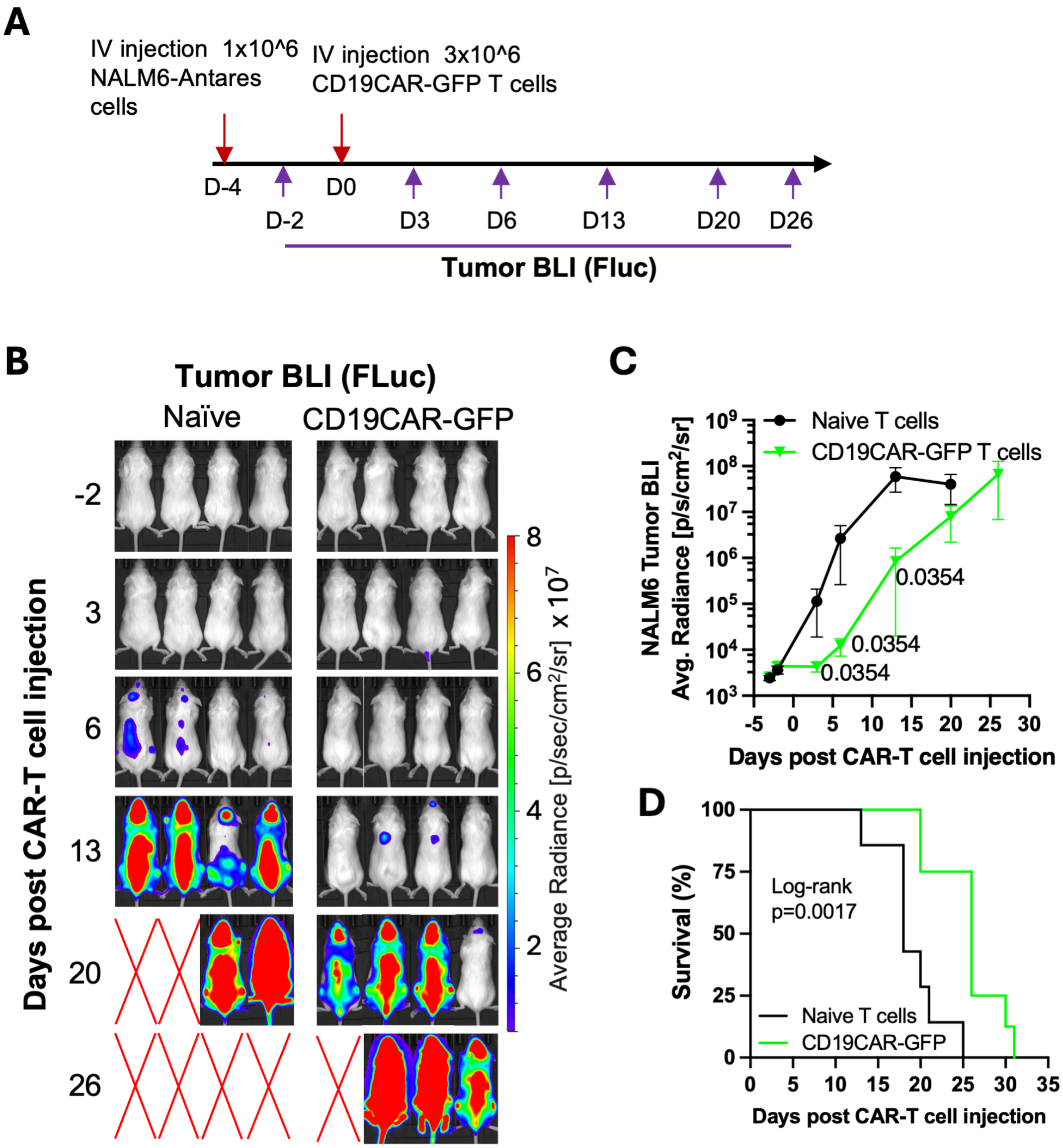

Figure S3. In vivo validation of CD19CAR-GFP T cell therapeutic efficacy in a NALM6 leukemia xenograft model.

A) Schematic of the experimental timeline using NALM6 ($dTomato^+/FLuc^+$) cells and CD19CAR-GFP T cell treatment. **B)** Representative bioluminescence imaging (BLI) of tumor burden over time, visualizing $FLuc^+$ NALM6 cells. **C)** Quantification of whole-body BLI signal. Data were log-transformed and analyzed using repeated measures two-way ANOVA with Geisser-Greenhouse correction, followed by Holm-Sidak's multiple

comparisons test. **D)** Kaplan-Meier survival analysis comparing mice treated with naïve T cells (n=7) versus CD19CAR-GFP T cells (n=8); Log-rank test. Statistical significance indicated.

Figure S4

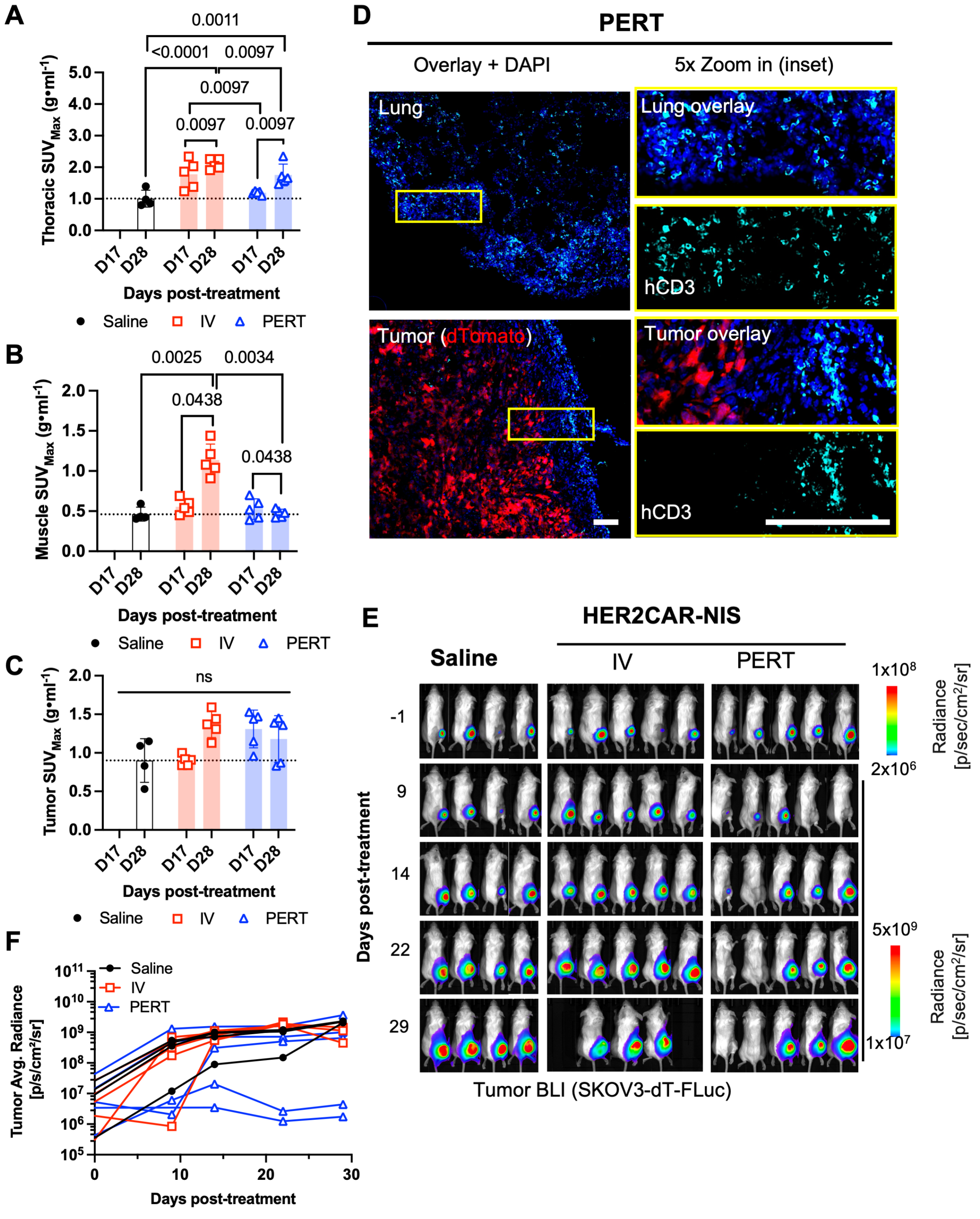

Figure S4. ***In vivo*** **[^{18}F]-TFB PET SUV$_{Max}$ imaging data and BLI monitoring of subcutaneous ovarian tumors following HER2CAR-NIS T cell therapy.**

A–C) Maximum standardized uptake value (SUV_{Max}) measured in thoracic (A), muscle (right hind limb, B), and tumor regions (C), respectively. Data analyzed using two-way ANOVA with Holm–Šidák's multiple comparisons test. Dotted line indicates the average SUV_{Max} of saline-treated controls. Symbols represent individual animals. **D)** Representative close-up of immunofluorescence staining showing $CD3^{+}$ T cells (cyan) in lung and tumor sections from a PERT-treated mouse. Tumor cells express dTomato (red); nuclei counterstained with DAPI (blue). Scale bar = 100 µm. **E)** BLI images depicting SKOV3-ip1 ($dTomato^{+}/FLuc^{+}$) tumor burden. **F)** Spaghetti plots of tumor BLI per mouse following saline, intravenous (IV) or peritumoral (PERT) HER2CAR-NIS T cell administration.

Figure S5.

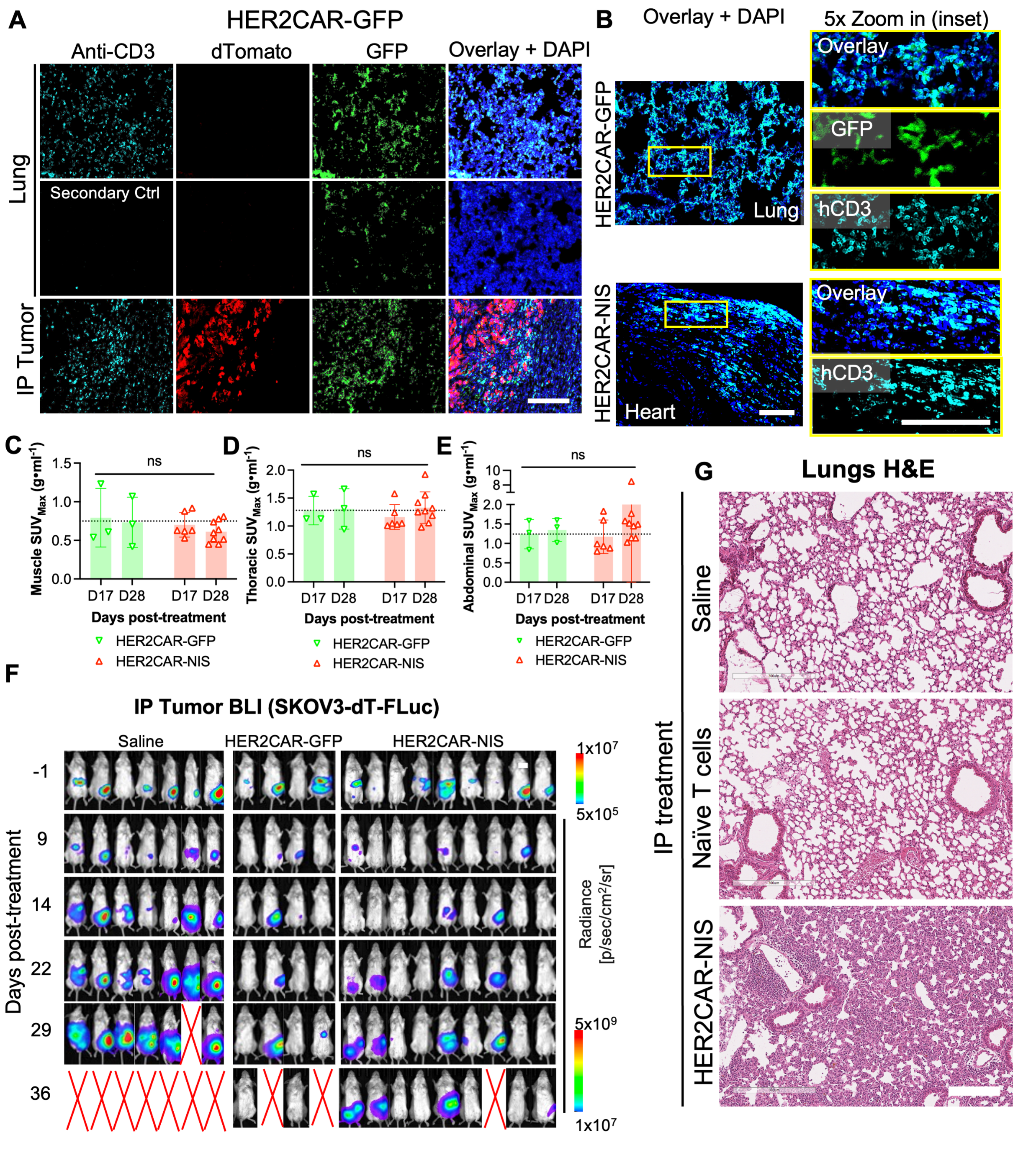

Figure S5. Imaging and histological analysis of HER2CAR-GFP and HER2CAR-NIS T cell locoregional therapy *in vivo*.

A) Immunofluorescence staining of lung and intraperitoneal (IP) tumor tissues shows co-localization of $CD3^+$ T cell infiltration and GFP reporter gene expression in the HER2CAR-

GFP cohort. In red, dTomato+ SKOV3-ip1 tumor cells. Overlay images include DAPI nuclear counterstain. **B)** T cell infiltration, as determined by GFP and/or CD3 fluorescence, in the lungs and pericardial tissue of HER2CAR-GFP and -NIS treated mice. **C–E)** Quantitative PET imaging analysis of [^{18}F]-TFB uptake reveals elevated SUV_{Max} values in the abdominal cavity of HER2CAR-NIS mice compared to HER2CAR-GFP controls at Day 28 post-treatment, consistent with NIS-mediated tracer accumulation. Dotted line indicates the average SUV_{Max} of HER2CAR-GFP-treated controls. Symbols represent individual animals. Differences were not statistically significant (ns). Data were analyzed using a non-parametric Mann–Whitney test with Holm–Šidák correction for multiple comparisons. **F)** Longitudinal bioluminescence imaging (BLI) of SKOV3-dT-FLuc IP tumors showing differential tumor burden across treatment groups (Saline, HER2CAR-GFP, HER2CAR-NIS). **G)** H&E staining of lung tissues highlights histopathological changes following cell therapy, with increased lung remodeling in HER2CAR-NIS-treated mice not seen in Naïve T cell- or Saline-treated mice. Statistical significance indicated; ns, not significant. Scale bars = 200 μm
